# Genome Editing and Protoplast Regeneration to Study Plant–Pathogen Interactions in the Model Plant *Nicotiana benthamiana*

**DOI:** 10.3389/fgeed.2020.627803

**Published:** 2021-01-21

**Authors:** Chen-Tran Hsu, Wen-Chi Lee, Yu-Jung Cheng, Yu-Hsuan Yuan, Fu-Hui Wu, Choun-Sea Lin

**Affiliations:** Agricultural Biotechnology Research Center, Academia Sinica, Taipei, Taiwan

**Keywords:** FnCas12a, nCas9-Target-AID, RDR6, SaCas9, SGS3, SpCas9

## Abstract

Biotic diseases cause substantial agricultural losses annually, spurring research into plant pathogens and strategies to mitigate them. *Nicotiana benthamiana* is a commonly used model plant for studying plant–pathogen interactions because it is host to numerous plant pathogens and because many research tools are available for this species. The clustered regularly interspaced short palindromic repeats (CRISPR) system is one of several powerful tools available for targeted gene editing, a crucial strategy for analyzing gene function. Here, we demonstrate the use of various CRISPR-associated (Cas) proteins for gene editing of *N. benthamiana* protoplasts, including *Staphylococcus aureus* Cas9 (SaCas9), *Streptococcus pyogenes* Cas9 (SpCas9), *Francisella novicida* Cas12a (FnCas12a), and nCas9-activation-induced cytidine deaminase (nCas9-Target-AID). We successfully mutated *Phytoene Desaturase* (*PDS*) and *Ethylene Receptor 1* (*ETR1*) and the disease-associated genes *RNA-Dependent RNA Polymerase 6* (*RDR6*), and *Suppressor of Gene Silencing 3* (*SGS3*), and confirmed that the mutated alleles were transmitted to progeny. *sgs3* mutants showed the expected phenotype, including absence of *trans-acting siRNA3* (*TAS3*) siRNA and abundant expression of the GFP reporter. Progeny of both *sgs3* and *rdr6* null mutants were sterile. Our analysis of the phenotypes of the regenerated progeny indicated that except for the predicted phenotypes, they grew normally, with no unexpected traits. These results confirmed the utility of gene editing followed by protoplast regeneration in *N. benthamiana*. We also developed a method for *in vitro* flowering and seed production in *N. benthamiana*, allowing the regenerants to produce progeny *in vitro* without environmental constraints.

## Introduction

*Nicotiana benthamiana* is a host to many plant pathogens, especially viruses, and is widely used to study plant–pathogen interactions (Goodin et al., 2008). Many tools for functional genomics are available for this species, including viral vectors, RNA interference (RNAi), ethyl methanesulfonate mutagenesis, agroinfiltration, protoplast transfection, and *Agrobacterium*-mediated stable transformation. These tools are useful for research in genomics, biochemistry, metabolomics, cell biology, and pathology, as well as other topics in agriculture (Derevnina et al., 2019).

Notwithstanding its many advantages, the fact that *N. benthamiana* is allotetraploid, with a very large genome (3.1 Gb) (Bombarely et al., 2012), makes it difficult to edit the genome of this plant and to obtain mutants for plant biological and gene functional studies. We chose to address this problem by using the powerful genome-editing tool CRISPR-Cas (clustered regularly interspaced short palindromic repeats-CRISPR-associated protein). CRISPR-Cas core technology involves programmable DNA cleavage by the Cas protein at DNA sites specified by the targeting sequence in a guide RNA (gRNA; review by Yue et al., 2020). The use of CRISPR-Cas has greatly accelerated plant research and crop breeding in recent years (Li et al., 2013; Nekrasov et al., 2013; Shan et al., 2013; Li and Xia, 2020; Yue et al., 2020).

Most genome editing studies in plants, including *N. benthamiana*, have involved Agrobacterium-mediated stable transformation to deliver DNA into target cells in order to express Cas protein and gRNA. However, mutant plants derived from Agrobacterium-mediated transformation could be considered genetically modified organisms (GMOs), especially for vegetatively propagated crops in which the transgenes cannot be removed from the genome by crossing. In dicots, however, most transformants are chimeric, and the edited allele cannot be transmitted to the progeny when the edited cells exist only in vegetative organs. Thus, as a less controversial alternative, plasmids encoding the Cas and gRNA sequences or pre-assembled Cas:gRNA ribonucleoprotein complexes (RNPs) can be delivered directly into protoplasts using transient transfection. Because the protoplast is a single cell, once gene editing has been performed, the entire regenerant derived from this edited protoplast will contain the same edited gene (Woo et al., 2015; Lin et al., 2018; Hsu et al., 2019). Although a similar type of delivery can also be achieved by particle bombardment, polyethylene glycol (PEG)-mediated protoplast transfection offers high transfection efficiency and high viability for robust gene editing while generating recombinant-DNA-free plants to circumvent GMO issues (Woo et al., 2015; Andersson et al., 2018; Lin et al., 2018).

The main bottleneck of this strategy, however, is protoplast regeneration. We previously established a protoplast regeneration system and a CRISPR-Cas gene editing system for polyploid tobacco (*N. tabacum*) (Lin et al., 2018; Hsu et al., 2019). Here we report a simple, highly robust protocol for streamlined CRISPR-mediated genome editing in *N. benthamiana*. This protocol, together with CRISPR genome editing and improved genomics resources, ushers in a new era of forward and reverse genetic analyses of this valuable model plant system.

## Materials and Methods

### Plant Materials

Sterile *N. benthamiana* plantlets were propagated by cutting and grown in half-strength Murashige and Skoog (1/2 MS) medium supplemented with 30 mg/L sucrose and 1% agar, pH 5.7. These plantlets were incubated in a 26°C culture room (12 h light /12 h dark cycle) with a light density of 75 μmol m^−2^ s^−1^. The plantlets were subcutlured into fresh medium every month. For comparison with the seedlings derived from protoplasts and seed propagation, seeds were sown in 3-inch pots with peat moss, vermiculite and perlite in a ratio of 5:1:1. Each treatment had five repeats.

### Protoplast Isolation and Transfection

The protoplast isolation and transfection followed our previously published method with minor modification (Hsu et al., 2019). The protoplasts were isolated from the mature leaves of *in vitro* plantlets. Five to seven leaves (about 0.2–0.25 g) were used for 10^6^ protoplast isolation. These leaves were put into a 6-cm glass petri dish with 10 ml digestion solution (1/4 MS liquid medium containing 1% cellulose, 0.5% Macerozyme, 3% sucrose and 0.4 M mannitol, pH 5.7) and cut into 0.5 cm-wide strips. The solution was incubated at room temperature in the dark overnight. The digested solution was diluted with 10 ml W5 (154 mM NaCl, 125 mM CaCl_2_, 5 mM KCl, 2 mM MES, and 5 mM glucose) solution and filtered by 40 μm nylon mesh. The solution was centrifuged at low-speed (200 × g) for 3 min to collect the protoplasts. The protoplasts were purified with 20% sucrose solution and washed in W5 solution three times. The protoplasts were transferred to a transfection buffer (1/2 MS solution supplemented with 3% sucrose, 0.4 M mannitol, 1 mg/L naphthaleneacetic acid (NAA), and 0.3 mg/L kinetin, 5 mM MES, pH 5.7) and the cell concentration was adjusted to 3 × 10^5^/mL.

The protoplasts were transfected with plasmids by PEG-mediated transfection (Woo et al., 2015; Lin et al., 2018). CRISPR reagent DNA (40 μg in 40 μl) was added to 400 μl (1.2 × 10^5^ protoplasts) and mixed carefully. Then the same volume of PEG solution was added and mixed, then left to stand for 30 min. To end the reaction, 3 ml of W5 was added and mixed well. The transfected protoplasts were collected by centrifugation at 200 × g for 3 min. The protoplasts were washed in 3 ml of W5 by centrifugation at 200 × g for 3 min.

### Plasmids

Several target sites in *N. benthamiana* whose editing efficiencies have been confirmed in *N. tabacum* (Hsu et al., 2019) as well as new constructions were used in this study. The following Cas proteins and target genes were tested in *N. benthamiana*:

1. SaCas9: The binary plasmid (gPDA_Sa) was published by Kaya et al. (2016). The target gene is *Phytoene Desaturase 1 (NbPDS-1)*, the target site is TTGCGATGCCTAACAAGCCAG.
2. FnCas12a: The binary plasmids (*crNtPDS-1* and *crNtPDS-2*) were published by Endo et al. (2016). Target genes are *NbPDS-1* and *NbPDS-2*, and the target sites are TCATCCAGTCCTTAACACTTAAAC*(crNtPDS-1)*, and ACATGGCAATGAACACCTCATCTG *(crNtPDS-1)*.
3. nCas9-Target-AID: The plasmid (pDicAID_nCas9-PmCDA-2A-NptII_ETR) is published in Shimatani et al. (2017) (Addgene ID: 91695). The target genes are *NbETR1-1* and *NbETR1-2*. The target site is TGCACAAGAACCCATCTATA.
4. SpCas9: the vector commonly used for dicot transformation (pYLCRISPR/Cas9P35S-N) is used (Ma et al., 2015). The target genes were *RNA-dependent RNA Polymerase 6 (NbRDR6-1* and *NbRDR6-2*), and *Suppressor of Gene Silencing 3 (NbSGS3-1* and *NbSGS3-2*). For convenience, to validate the presence and efficiency of the mutations, double sgRNAs were present in a single construct for each gene *(NbSGS3*: AAGCAGTGCTGGGAAGCAAT, CTCATGCCACGATGGCCTTG; *NbRDR6*: GCCATGGCCTTCTCAAAGCT, GCAGTTCTATAGAAAACCAA). The sgRNAs were cloned into vectors.

### Protoplast Regeneration

**Figure 1:**
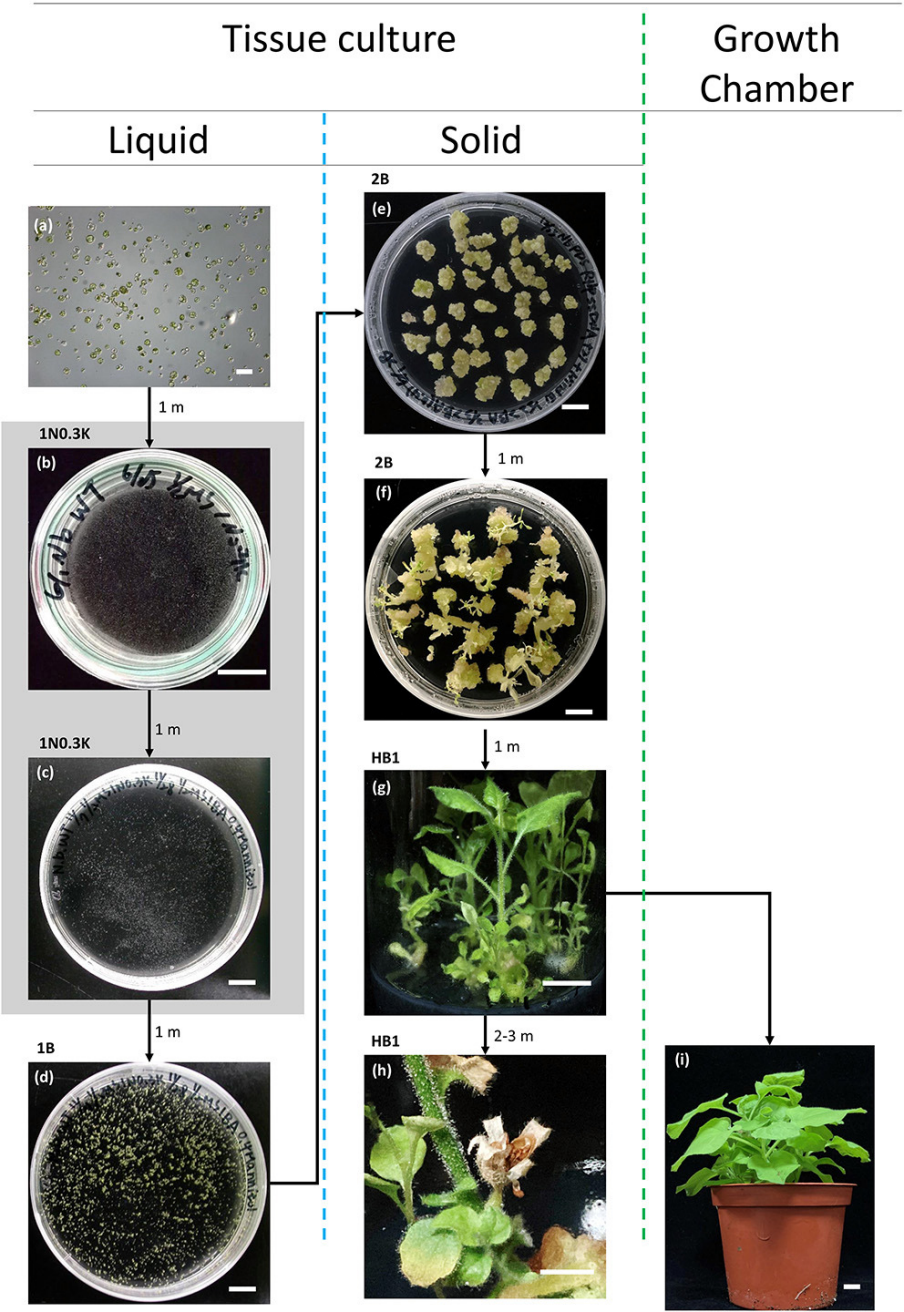
Protoplast regeneration in *N. benthamiana*. The gray background indicates that the explants were incubated in the dark. All liquid media were supplemented with 0.4 M mannitol. The abbreviated names of the media are shown in the top left corner of the images. 1N0.3K: 1/2 MS basal medium supplemented with 1 mg/L NAA and 0.3 mg/L kinetin. 1B: ½ MS basal medium supplemented with 1 mg/L BA and 20 g/L sucrose. All solid media were solidified with 7 g/L Phytagel. 2B: 1/2 MS basal medium supplemented with 2 mg/L BA. HB1: 3 g/L Hyponex No. 1 (N:P:K = 7:6:19), 2 g/L tryptone, 20 g/L sucrose, and 1 g/L activated charcoal. **(a)** Transfected mesophyll protoplasts. Bar = 200 μm. **(b)** Microcallus formation after 1 month of incubation in 1N0.3K. Bar = 1 cm. **(c)** Microcallus amplification. **(d)** Microcalli transferred to 1B medium and incubated in the light. Bar = 1 cm. **(e)** Callus amplification in 2B. Bar = 1 cm. **(f)** Shoot formation in 2B. Bar = 1 cm. **(g)** Shoots incubated in HB1 for root formation. **(h)** *In vitro* seed formation. Bar = 5 mm. **(i)** Plantlet incubated in a growth chamber. Bar = 1 cm.

Pooled protoplast DNA was used as a template to amplify the target genes for validation by sequencing. The putatively edited protoplasts were transferred to 5 cm diameter Petri dishes containing 3 ml 1/2 MS liquid medium supplemented with 3% sucrose, 0.4 M mannitol, 1 mg/L NAA, and 0.3 mg/L kinetin (1N0.3K) for plant regeneration. Callus formation occurred using protoplasts after 1 month of incubation in the dark. The calluses were subcultured in 9 cm diameter Petri dishes containing fresh medium with 1 mg/L 6-benzylaminopurine (1B) for 3–4-weeks in the light. Calluses that had turned green were then transferred to solid medium containing the same plant growth regulators. The explants were subcultured every 4 weeks until shoots formed after several subcultures. The shoots were subcultured in solid root medium (HB1: 3 g/L Hyponex No. 1, 2 g/L tryptone, 20 g/L sucrose, 1 g/L activated charcoal, 10 g/L Agar, pH 5.2). Adventitious roots formed at the bottoms of the containers (Figure 1).

### Genotype Analysis of Regenerated Plants

Two pairs of primers were designed to amplify the sgRNA-targeted DNA region for each target gene. PCR conditions were 94°C for 5 min, 35 cycles of denaturing (94°C for 30 s), annealing (55°C for 30 s), and polymerization (72°C for 30 s), followed by an extension reaction at 72°C for 5 min. The PCR product was sequenced by the Sanger method to determine the mutagenesis. The multiple sequences derived from mutated regenerated plants were separated using Poly Peak Parser (http://yosttools.genetics.utah.edu/PolyPeakParser/; Hill et al., 2014) or further confirmed by sequential T/A cloning and sequencing.

## Results

### *N. benthamiana* Protoplast Regeneration

For protoplast regeneration, we placed protoplasts isolated from the leaves of *in vitro-*grown shoots (Figure 1a) in 1N0.3K liquid medium, incubated them in the dark for 1 month (Figure 1b), and then transferred them to fresh 1N0.3K medium and incubated them in the dark for another month (Figure 1c). Unlike in our previous method described for tobacco (Lin et al., 2018), we incubated *N. benthamiana* calluses directly in liquid 1B medium in light without embedding (Figure 1d). This step avoids the difficulty associated with embedding; however, it has the disadvantage that the calluses stick together and sometimes cannot be distinguished. After 1 month, we transferred the calluses larger than 3 mm to 1B solid medium and incubated them in light (Figure 1e). After several subcultures, shoots formed on the surface of the calluses (Figure 1f); this took more time for *N. benthamiana* than it does for *N. tabacum* (Lin et al., 2018). We then subcultured the shoots in solid HB1 medium and observed that adventitious roots formed without the need for plant growth regulators (Figure 1g). These plants could be further incubated successfully in test tubes, where they flowered and produced seeds (Figure 1h), or were transferred to a growth chamber for further growth (Figure 1i). The time required from protoplast isolation to regeneration was ~4–6 months.

**Figure 2:**
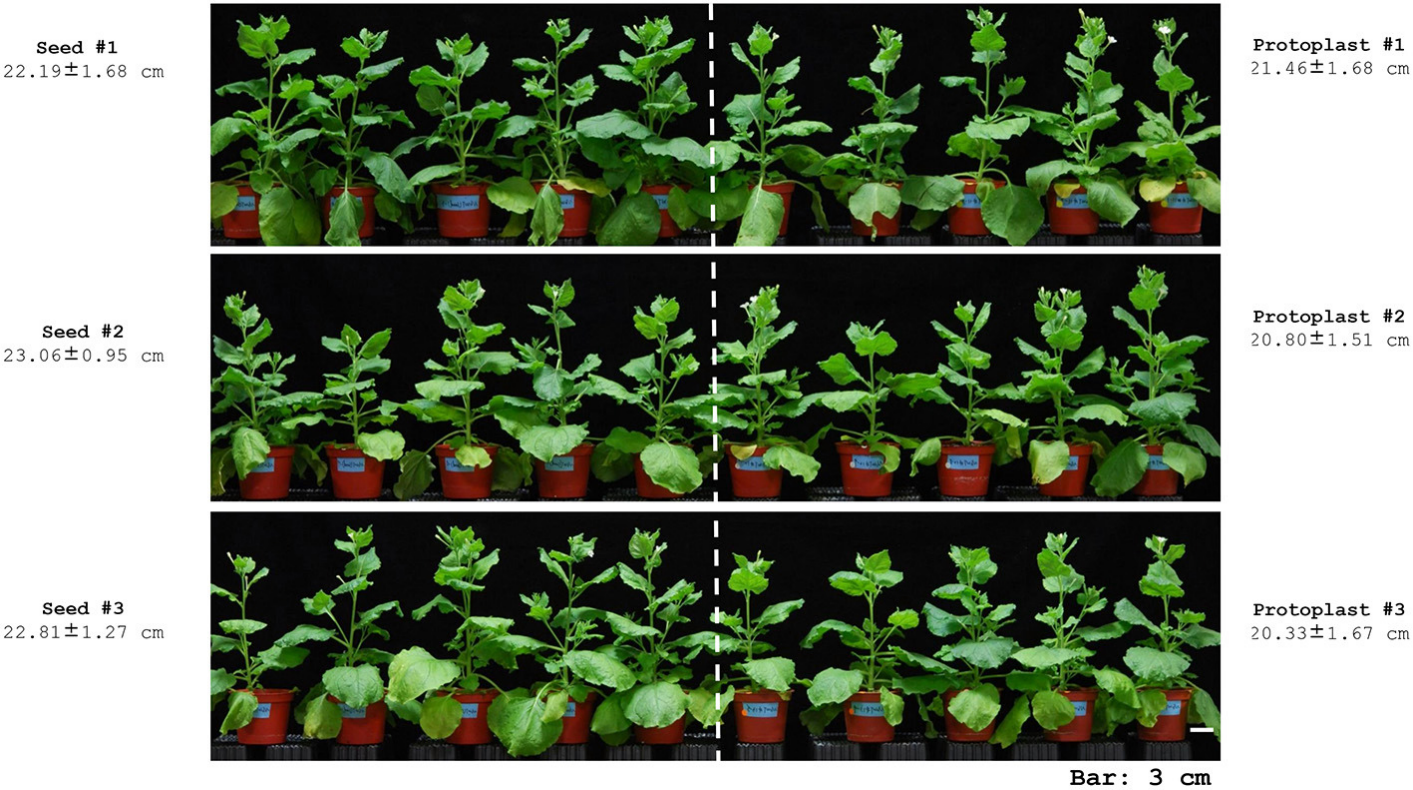
Phenotypes of seed-derived seedlings and seedlings regenerated from protoplasts.

To look for unexpected phenotypes in the regenerants, we randomly selected three regenerated plants (protoplasts #1, #2, and #3) and harvested their seeds. We grew the progeny in a growth chamber for 40 days and compared them to seedlings derived from seed propagation (seed #1, #2, and #3). We observed no significant differences in plant height between the regenerants and seed-derived plants (Figure 2). All plants flowered and produced seeds normally.

### CRISPR Efficiency

**Figure 3:**
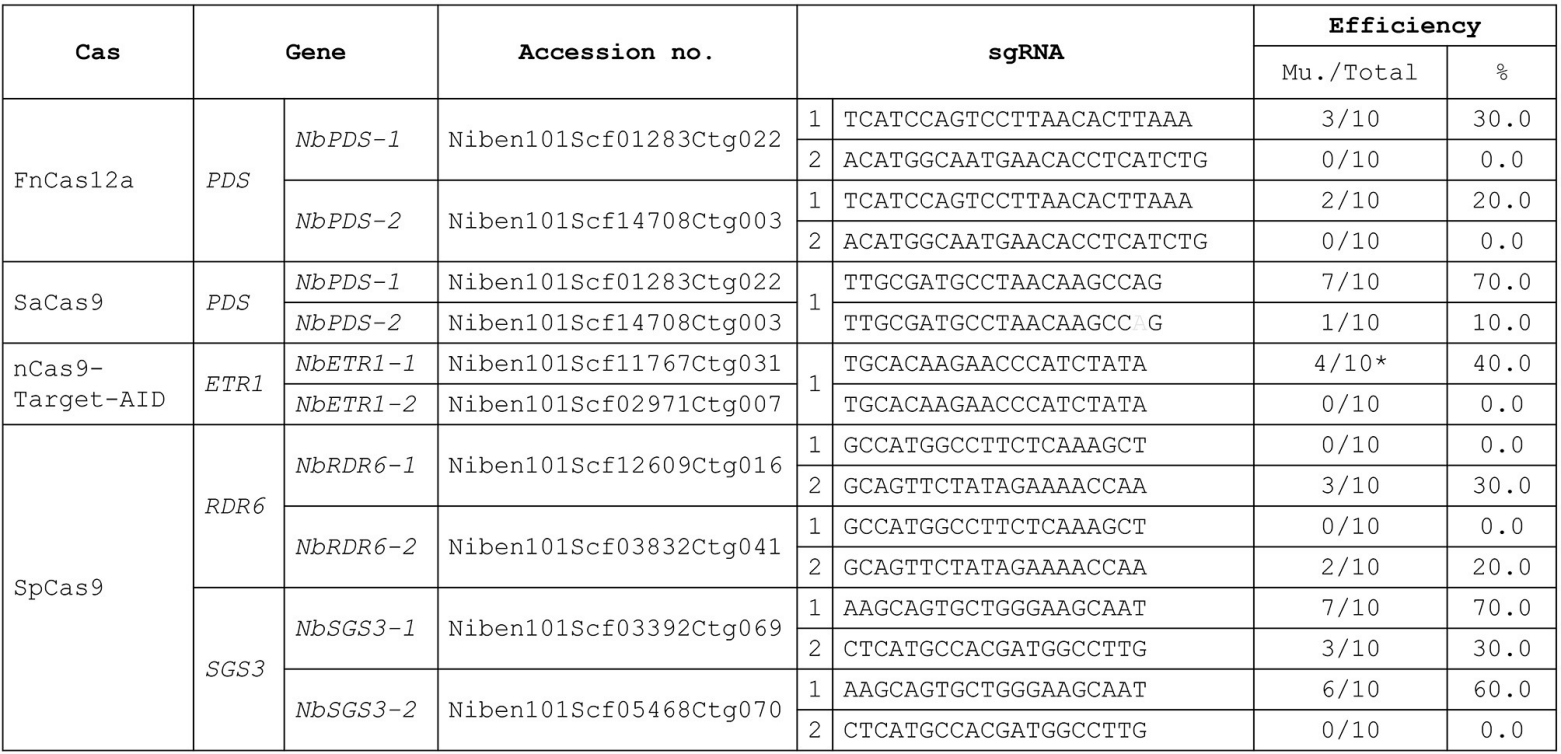
Target mutagenesis efficiencies. Mu, mutants. Gray: sgRNA mismatch. *: mutation. There were 10 regenerated plants (Total) analysis in each transfection. The mutants were confirmed by Sanger sequencing. % = No. of mutants/total no. of regenerated plants analyzed) X 100.

To demonstrate that this protocol can be used for CRISPR-mediated gene editing, we performed protoplast transfections using plasmids previously shown to be effective in *N. tabacum* (Hsu et al., 2019). We successfully used SaCas9, FnCas12a, and nCas9-Target-AID to obtain regenerated plants for target gene editing using this protocol. The efficiency of *N. benthamiana* transformation was similar to that of *N. tabacum* (Figure 3, Hsu et al., 2019). As in *N. tabacum*, three different Cas proteins were successfully used to edit different target genes simultaneously in a single *N. benthamiana* protoplast. The target site of SaCas9 has a mismatch in *NbPDS-2*, and there was 10.0% off-target editing of *NbPDS-2* (Figure 3). In nCas9-Target-AID, we only observed mutation and no C to T editing regenerant was obtained. We designed *NbSGS3* and *NbRDR6* sgRNAs that can be used in both *N. tabacum* and *N. benthamiana* and introduced them into the SpCas9 plasmid. The regeneration results indicated that, except for sgRNA 2 in *RDR6*, these sgRNAs had target mutagenesis efficiencies in *N. benthamiana* (Figure 3) and *N. tabacum* (data not shown).

### Phenotypes of Regenerants Following Targeted Editing

For the *PDS* gene study, we used SaCas9 and FnCas12a for targeted mutagenesis. When we used SaCas9 alone, since there was a mismatch in *NbPDS-2* (Niben101Scf14708Ctg003), we obtained no *NbPDS-1* and *NbPDS-2* double knock out mutants, and found *nbpds-1* null mutants with wild-type or heterozygous *NbPDS-2*. These mutants did not appear albino, the usual phenotype for this mutant, because *NbPDS-2* was still functional, unlike our previous findings with *N. tabacum* in which double knock out mutants were obtained using the same plasmid (Lin et al., 2018). Although the mutagenesis efficiency was low, because the target sequences of *NbPDS-1* and *NbPDS-2* are identical, we still obtained albino *nbpds-1 nbpds-1/nbpds-2 nbpds-2* double null mutants in regenerants derived from FnCas12a-mediated transfection. We also obtained *NbPDS-1 nbpds-1/nbpds-2 nbpds-2* heterozygous T_0_ plants when the protoplasts were transfected with three plasmids (SaCas9, FnCas12a, and Target-AID). Albino mutants were detected in the T_1_ offspring, and their proportions and genotypes were as expected.

**Figure 4:**
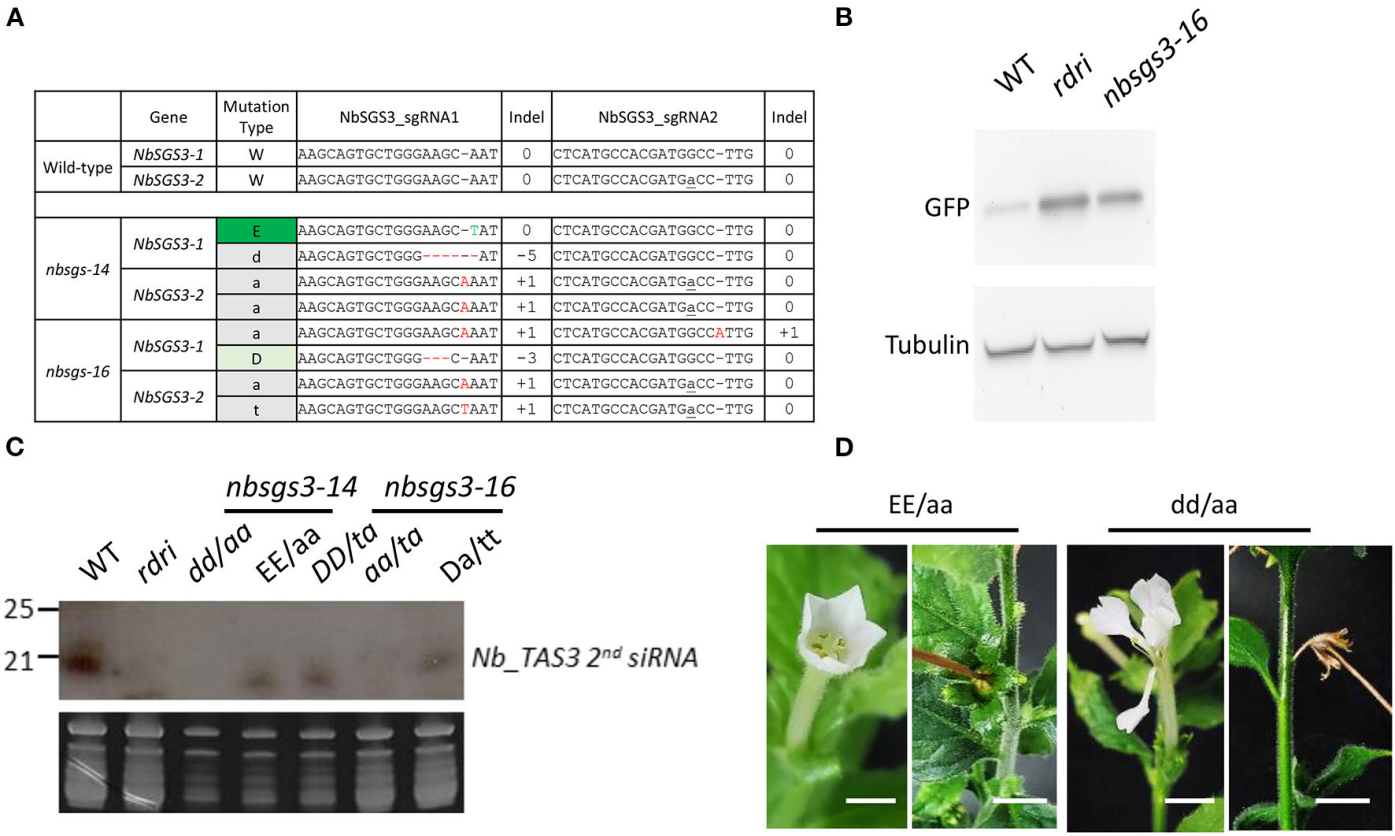
Mutants regenerated from CRISPR-Cas-edited *Nicotiana benthamiana* protoplasts. **(A)** Genotypes of F_1_ *N. benthamiana sgs3* mutants used for small RNA analysis. *NbSGS3-1*: Niben101Scf03392Ctg069; *NbSGS3-2*: Niben101Scf05468Ctg070. Red -: deleted nucleotide. Letter in gray: inserted nucleotide. Letter in green: edited nucleotide. **(B)** Five-week-old *N. benthamiana* plants subjected to Agrobacterium-mediated transient infiltration with 1 OD Agrobacterium cultures (OD_600_ = 1) harboring binary vector with *Green fluorescence protein* (*GFP*) driven by the cauliflower mosaic virus *35S* promoter. Leaves were harvested 3 d after infiltration. GFP and tubulin levels were analyzed by immunoblot analysis. **(C)** RNA gel blot analysis of the progeny of the *Nbsgs* mutants. WT, wild type; *rdri*, RNAi line of *NbRDR6*; *D*, 3-bp deletion. T insertion. **(D)** The progeny of *Nbsgs3-14*. Uppercase letters: in frame; lowercase letters: out of frame. *E*: base editing. *a*: A insertion. *d*: 5-bp deletion. Bar = 5 mm.

The homozygous *nbrdr6-1 nbrdr6-2* double mutant derived from protoplast regeneration (*nbrdr6*#C13) was sterile, as are genome-edited mutants obtained via Agrobacterium-mediated transformation (Ludman and Fátyol, 2019; Matsuo and Atsumi, 2019), because they fail to produce seeds. Interestingly, two *N. benthamiana sgs3-1 sgs3-2* biallelic mutants (*nbsgs3*-14 and *nbsgs3*-16) both produced seeds. We identified four editing “scars” in *nbsgs3*-14 (Figure 4A): a 1-bp substitution (E) and a 1-bp insertion (a) in *NbSGS3-1*, and a 5-bp deletion (d) and a 1-bp insertion in *NbSGS3-2* (Figure 4B). Progeny with the EE/aa genotype could produce seeds, but dd/aa plants bore abnormal flowers that failed to produce fertile seeds (Figure 4C), as did the *nbsgs3-14* aa/at progeny. All *nbsgs3-14* progeny, regardless of their genotype, exhibited lower expression of the trans-acting secondary siRNA TAS3 than the wild type (Figure 4D). In sterile progeny (dd/aa in *nbsgs3-14*, aa/at in *nbsgs3-16*), no TAS3 siRNA was detected. These results indicate that RNA silencing was aberrant in the *nbsgs3* mutants. *RDR6* and *SGS3* function in RNA silencing by reducing foreign gene expression. Similar to the enhanced transgene expression observed in Agrobacterium-infiltrated *N. benthamiana rdr6* mutants (Ludman and Fátyol, 2019; Matsuo and Atsumi, 2019), GFP accumulated to higher levels in *nbsgs3-16* than in wild type (Figure 4B).

### *In vitro* Flowering

**Figure 5:**
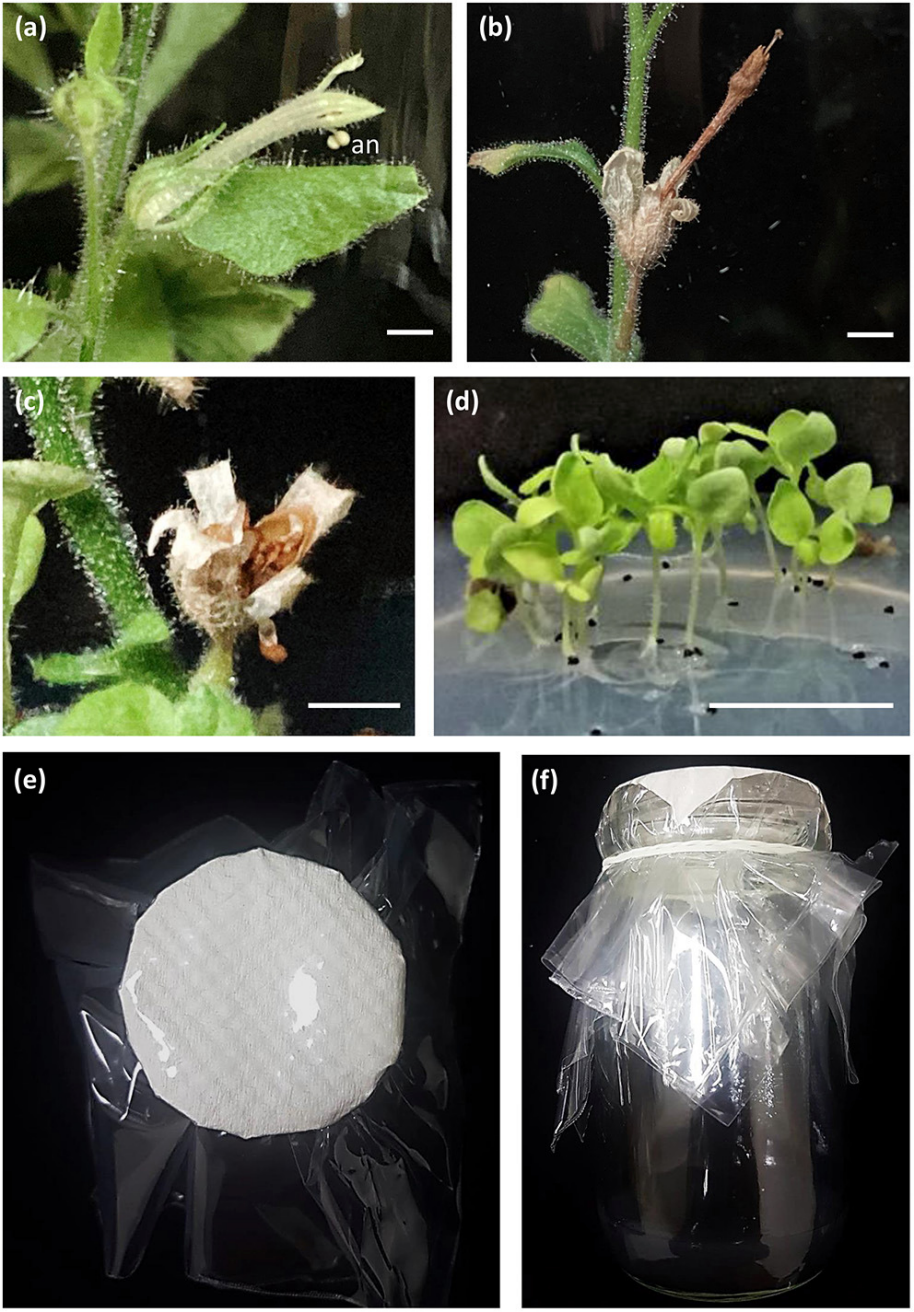
*In vitro* flowering and seed production in *N. benthamiana*. **(a)** *In vitro* flower. an: anther. Bar = 5 mm. **(b)** Mature fruit. Bar = 0.5 mm. **(c)** Open capsule. Bar = 0.5 mm. **(d)** Seedlings derived from *in vitro* seeds. Bar = 1 cm. **(e)** Bottle lip sealed with our newly developed sealing material. Bar = 1 cm. **(f)** Subculture in HB1 medium. Bar = 1 cm.

We incubated *N. benthamiana* plants regenerated from protoplasts in the same medium used for *in vitro* flowering of the orchid *Erycina pusilla* (Chiu et al., 2011). The *N. benthamiana* regenerants were able to flower (Figure 5a) and produce seeds (Figure 5b) *in vitro*. The seeds matured normally (Figure 5c) and germinated (Figure 5d). To investigate whether this medium can be widely used, we also incubated plants regenerated from protoplasts of other species in the same medium, including protoplasts from tobacco, broccoli, cauliflower, Arabidopsis, and rapid cycling *Brassica oleracea*. The tobacco, broccoli, and cauliflower plants did not flower *in vitro*, whereas the Arabidopsis and rapid cycling *Brassica oleracea* plants flowered but failed to produce seeds.

To increase seed production, we attempted to reduce the humidity inside the flask. For this purpose, we designed a new sealing material. A small piece of paper larger than the diameter of the bottle lip was placed inside two pieces of plastic film (Figure 5e), sealed over the lip, tied with a rubber band (Figure 5f), and sterilized by autoclaving. *N. benthamiana* produced seeds normally under these conditions, whereas the other species were still unable to produce seeds.

## Discussion

During this study, we determined that protoplasts derived from *N. tabacum* are easier to regenerate than protoplasts from *N. benthamiana*. The bottleneck in regenerating *N. benthamiana* plants occurs during the step from callus to shoot formation: whereas most calluses derived from *N. tabacum* can be regenerated into shoots after a single subculture, *N. benthamiana* requires two or three successive subcultures before shoots form, and the proportion of shoots that form in each subculture step is unpredictable. Nonetheless, *N. benthamiana* still has many advantages. Compared to *N. tabacum, N. benthamiana* requires less space, and it can flower all-year round, whereas in subtropical regions, *N. tabacum* cannot grow and produce flowers in the greenhouse in summer.

Protoplast regeneration has been used since 2016 for transcription activator-like effector nucleases (TALEN)-mediated gene editing (Li et al., 2016). Although this strategy has many advantages with regard to gene editing procedures, it is often avoided. Instead, protoplasts have mainly been used for protoplast fusion and mutation. Furthermore, protoplast regeneration is thought to result in many unanticipated mutations. Indeed, a study involving whole-genome sequencing of potato plants regenerated from protoplasts suggested that protoplast regeneration can cause numerous mutations and even chromosome rearrangements (Fossi et al., 2019). Because the tobacco genome is so large, we have not yet sequenced the entire genomes of gene-edited *N. benthamiana* regenerants, and thus we have not directly investigated their levels of mutations. However, our comparison of regenerated plants with seed-propagated offspring indicated that this protocol does not produce plants with phenotypic differences from the wild type.

In fact, unexpected mutations can occur in any tissue culture process and even under natural conditions (Lin and Chang, 1998; Yue et al., 2020). In crop breeding, even if mutations occur, desired offspring can be identified through selection from a wide range of gene-edited regenerated plants, without the ethical problems associated with the human application of genome editing (Tang et al., 2019). In plant research, the problem of unexpected mutations could be resolved by generating multiple mutations of the same gene, such as in Arabidopsis and rice knockout lines, or by transferring the edited gene to a wild-type plant by crossing.

Given concerns about the use of genetically modified crops, it is important to be able to produce genetically-edited crops without introducing foreign genes. In particular, although transgenes introduced via stable transformation can be removed from many plants through crossing, this is not the case for the many important crops that are propagated asexually, such as potato. The delivery of CRISPR reagents into cells by transient transfection, however, is widely regarded as transgene-free gene editing. Using protoplast regeneration, RNPs (Woo et al., 2015) or plasmids (Lin et al., 2018; Hsu et al., 2019) can be used as CRISPR reagents for transgene-free gene editing; this is the main reason that we use protoplast regeneration for gene editing of crops. In our experience, both RNPs and plasmids are effective for gene editing. When choosing gene-editing reagents, if no documented gene target sites are available to confirm editing efficiency, we use plasmids, which allow us to use multiple targets at once and are relatively cost-effective. For target insertion, we strongly recommend using RNPs, as plasmids may act as donor DNAs.

In addition, RNPs can be used to validate novel Cas proteins when the expected results are not obtained using plasmids. Since our Cas protein was translated and RNP-confirmed *in vitro*, we directly introduced these RNPs into protoplasts to validate that this protein functions in the species of interest. We used RNPs to monitor the efficiency of Cas12a proteins in *N. benthamiana*. Cas12a has a high target mutagenesis efficiency in Poaceae (Li et al., 2019). In dicots, LbCas12a has a higher editing efficiency than AsCas12a in soybean and tobacco protoplasts when delivered as RNP molecules (Kim et al., 2017). Similarly, when tested in rice, Arabidopsis, and maize, LbCas12a but not AsCas12a successfully edited target genes when these nuclease plasmids were delivered into protoplasts (Kim et al., 2017; Malzahn et al., 2019).

More importantly, the efficiency of Cas12a proteins is temperature dependent (Malzahn et al., 2019). These enzymes have high activity at 37°C, the temperature used for human cell culture, whereas plant transformation is performed at ~28°C, a temperature at which LbCas12a activity is reduced (Moreno-Mateos et al., 2017; Malzahn et al., 2019). Hence, it is likely that the absence of edited plants was due to the lower temperature along with the lower overall activity of AsCas12a. In both *N. tabacum* and *N. benthamiana*, the efficiencies of FnCas12a when using plasmids are <10%. When FnCas12a and AsCas12a RNPs (which were confirmed to have cleavage activity *in vitro*) were used, target mutagenesis did not occur, even when we increased the amount of RNP, raised the temperature, or changed the medium composition. Therefore, we suggest that the low target mutagenesis efficiency of Cas12a is due not to the low expression of this protein but to intracellular conditions unsuitable for its activity.

Because transient transfection can deliver multiple plasmids into the same protoplast at one time, Cas protein and sgRNAs do not necessarily need to be encoded by the same vector. The Agrobacterium transformation vector we used in the current study is a low copy number vector, which makes plasmid DNA extraction more difficult. Because the DNA does not need to be inserted into the chromosome, there is no need to clone these genes into the T-DNA vector. In addition, protoplast transfection is highly efficient and does not require a selectable marker for screening. Thus, to refine our method, we could simplify the vector structure and use a high copy number vector for CRISPR-mediated protoplast transfection. Alternatively, we could co-transfect *in vitro* transcribed sgRNA with the overexpression Cas protein vector to reduce the labor involved in construction (Zhang et al., 2016). When designing sgRNAs, we will not only use software to predict the efficiencies of the sgRNAs, but also select the relative positions of sgRNAs that have been successful for other species. For example, sgRNAs that were designed by this strategy based on the *RDR6* and *SGS3* sgRNAs that are effective in *N. benthamiana* were also effective in *Solanum peruvianum*. If there are many effective sgRNA candidates, we will choose the one that can be used in the largest number of species to increase usage. *ETR1* sgRNA, which we used successfully in *N. benthamiana* and *N. tabacum*, is derived from tomato (Shimatani et al., 2017). Applying such a principle for sgRNA design to simultaneously induce mutations in multiple genes or gene families in heterozygous or polyploid plants is difficult because of mismatches. Because this is not a problem for off-target crops, it can instead be exploited for this type of multiplex gene mutation (Endo et al., 2015).

Most explants grow to the vegetative stage *in vitro*. By manipulating the medium and culture conditions, however, many plants can also be induced to flower *in vitro*. For example, bamboo has a juvenile period of several decades in the natural environment but can flower within 1 year in medium containing cytokinin (Lin and Chang, 1998). Plant species that can flower and be successfully pollinated, form fruit, and complete all growth stages *in vitro* are potentially good model plants for further study. For example, *E. pusilla*, which has these capabilities, serves as a model plant of the orchid family (Chiu et al., 2011). Here we demonstrated that *N. benthamiana* can bear fruit and produce seeds *in vitro* and that the use of HB1 medium and sealing film that we developed can increase fruiting and seed production. Although a speed breeding method has been developed to accelerate a plant's growth cycle and achieve year-round production (Ghosh et al., 2018), this method is quite expensive. It is important to develop an economical, space-saving method that can be used by all laboratories. The *in vitro* method developed in the study represents an alternative strategy for achieving this goal. However, this method cannot be used for all crops, an issue that will need to be addressed.

## Conclusions

Although various protocols have been published for Agrobacterium-mediated stable transformation or DNA-free plant genome editing of *N. benthamiana* using virally delivered CRISPR-Cas (Ma et al., 2020), these techniques pose several problems, including issues related to the regulation of transgenic crops and the production of genetic chimeras. Protoplast regeneration represents an alternative approach for high-efficiency gene editing that avoids these complications. With this method, no foreign DNA is integrated into the chromosomes, the regenerated plants are derived from single cells, and all of the edited alleles can be passed on to the offspring. We also used this procedure to transfer large amounts of donor DNA to increase the efficiency of target DNA insertion. We believe that this system and the resulting mutants represent excellent tools for researchers using *N. benthamiana* for crop pathogen-related research.

## Data Availability Statement

The original contributions presented in the study are included in the article/supplementary material, further inquiries can be directed to the corresponding author/s.

## Author Contributions

C-SL conceived and designed the experiments. C-TH, and Y-HY performed the CRISPR-Cas9 experiments. C-TH, Y-HY, and C-SL conducted the protoplast regeneration. C-TH, W-CL, Y-HY, and F-HW performed the molecular biology experiments and targeted mutagenesis analysis. W-CL performed small RNA Northern analysis and Western analysis. C-SL wrote the manuscript with input from all co-authors. All authors read and approved the final manuscript.

## Conflict of Interest

The authors declare that the research was conducted in the absence of any commercial or financial relationships that could be construed as a potential conflict of interest.
